# Measuring global health-related quality of life in children undergoing hematopoietic stem cell transplant: a longitudinal study

**DOI:** 10.1186/1477-7525-11-26

**Published:** 2013-02-26

**Authors:** Angie Mae Rodday, Norma Terrin, Susan K Parsons

**Affiliations:** 1 The Institute for Clinical Research and Health Policy Studies, Tufts Medical Center, Boston, MA, USA; 2 Graduate Program in Clinical and Translational Science, Sackler School of Graduate Biomedical Sciences, Tufts University, Boston, MA, USA; 3 Tufts University School of Medicine, Boston, MA, USA

**Keywords:** Quality of life, Pediatrics, Hematopoietic stem cell transplantation

## Abstract

**Background:**

Pediatric health-related quality of life (HRQL) measures explore multiple domains of HRQL. To ease administration, burden, and implementation, we created a 7-item unidimensional global HRQL scale for children. This paper evaluates the psychometric properties of the global HRQL scale in children undergoing hematopoietic stem cell transplant (HSCT) and describes the trajectory of global HRQL scores over the 12-month course following HSCT.

**Methods:**

As part of two longitudinal HSCT studies, HRQL was collected on 312 parent–child dyads using the Child Health Ratings Inventories. Parents of children aged 5–18 completed the pediatric global HRQL scale about their child and 117 adolescents completed the scale themselves. Psychometric properties were compared across both raters. Two repeated measures models were built to describe trajectories of (1) global HRQL for all children based on parent proxy report and (2) global HRQL for adolescents based on adolescent self-report and parent proxy report.

**Results:**

Internal consistency reliability was high for parent proxy report and adolescent self-report (Cronbach’s alpha 0.9, 0.8, respectively). Unidimensionality was verified using principal components analysis. Both models indicated decreased global HRQL in the presence of early complications related to HSCT and Model 1 further indicated decreased HRQL in the presence of later complications. Model 2 showed that parent proxies reported lower global HRQL scores than adolescent self-report.

**Conclusions:**

This study has demonstrated the unidimensionality and strong psychometric properties of a 7-item global HRQL scale in a sample of children undergoing HSCT. Despite its brevity, scale scores vary in clinically meaningful ways. Future applications of this scale are encouraged.

## 

The use of patient-reported and patient-centered outcomes, such as health-related quality of life (HRQL), in clinical and epidemiological research has been increasing in recent years and is expected to continue increasing with the creation of the Patient-Centered Outcomes Research Institute (PCORI), which funds comparative effectiveness studies that emphasize patient-centered outcomes [1]. Due to methodological considerations (e.g., cognitive development), the field of HRQL measurement in children and adolescents initially lagged behind the development and validation of generic and disease-specific HRQL measures for adults [2]. Over the past two decades, this gap has been closing; there are currently several generic and disease-specific HRQL measures for children and adolescents that use self-report and/or parent proxy report [3-9].

Nearly all of the health profile HRQL measures for adults and children contain multiple domains for measuring the multidimensional constructs of HRQL, such as physical functioning, mental health or emotional functioning, and role functioning [3-10]. Some measures, such as the PedsQL™ 4.0, produce a total score, while other measures report domain-specific scores or subscale scores. Only recently has a unidimensional domain of global HRQL for adults been developed as part of the Patient-Reported Outcome Measurement Information System (PROMIS) project [11], a US National Institutes of Health (NIH)-funded initiative that develops and validates domains to measure feelings, functions, and perceptions applicable to a range of chronic conditions (http://www.nihpromis.org). To date, there is currently no comparable global HRQL scale for use in pediatric populations.

Global HRQL scales have appeal over other multidimensional HRQL measures for several reasons: (1) decreased respondent burden, (2) increased ease of implementation, (3) increased simplicity associated with having a single HRQL score, and (4) potential to direct future research and evaluation. With decreased respondent burden and increased ease of implementation, global HRQL scales could be used in a larger number of research studies and could also be used more frequently in longitudinal studies as compared to longer, multidimensional HRQL measures. Rather than having several different HRQL domain scores, the simplicity of having a single global HRQL score also could result in increased use in research studies and could eventually lead to use in clinical practice. Finally, the global HRQL scale could serve as a screener for further evaluation. For example, those who score at the lower extreme of the scale may receive additional questions to further understand why their global HRQL is low, or they could be targeted for intervention.

The first aim of this paper is to assess the psychometric properties of a pediatric global HRQL scale from the Child Health Ratings Inventories (CHRIs) in a sample of children prior to undergoing hematopoietic stem cell transplant (HSCT). The second aim is to determine the trajectory of the global HRQL scale scores over a 12-month period among two subgroups: (1) “all children” based on parent proxy report and (2) “adolescents only” based on adolescent self-report and parent proxy report. We rely on a sample of 312 children undergoing HSCT and their parents, which offers a unique opportunity to assess the performance of this global HRQL scale and the trajectories over time during an intense clinical course that is expected to result in changing HRQL.

## Methods

### Participants

This analysis draws on two completed studies: (1) 165 pediatric HSCT recipients aged 5–18 and their parents who were both enrolled in the Journeys to Recovery (JTR) study, which described the 12-month HRQL trajectory following HSCT and (2) 198 pediatric HSCT recipients aged 0–18 years and their parents who were both enrolled in the HSCT-Comprehensive Health Enhancement Support Study (CHESS™), which was a randomized controlled trial of a web-based intervention designed to improve the health-related knowledge, skills, and quality of life of parents of children undergoing HSCT. Together, these studies were conducted at eight HSCT centers across the United States from 2003 to 2011. Eligibility included a working knowledge of English and having a parent/legal guardian who could consent on behalf of the child. Parental consent and age-appropriate assent were obtained. Both studies were approved by the institutional review board (IRB) at Tufts Medical Center and at each clinical site; IRB approval was also obtained to combine data from these studies. Because the HRQL measures used to create the outcome for this analysis were developed for use in children and adolescents aged 5–18, parent and child dyads were removed from the analysis if the child was less than five years old, resulting in a final sample size of 312.

### Measures

#### Child Health Ratings Inventories (CHRIs)

This validated measure has been used extensively in pediatric HSCT populations [4,5]. The parental version of the CHRIs-General module includes two principal sections. The first section elicits parental ratings of the child’s health and functioning, while the second section elicits the parent’s rating of his/her *own* functioning. The school-aged child version (ages 5–12) of the CHRIs-General module elicits the child’s rating of their own health and functioning using pictorial response sets, while the adolescent version (ages 13–18) is text-based with parallel wording to the parent version. Items from each of these versions form three domains of generic HRQL: physical, role, and emotional functioning. In addition to these three domains of HRQL, the CHRIs-General parent and adolescent versions also include an overview section that asks seven items relating to the child’s global HRQL (see Table 1 for item content), and is the focus of this paper. Unlike the items that form the three domains of HRQL, school-aged children do not complete questions about their personal appraisal of overall health and HRQL due to developmental reasons. All CHRIs items use an acute, 1-week recall period because of the rapidly changing health status of pediatric patients undergoing transplant. All response sets have five options. Using the half scale rule, which requires that at least half the items in a scale be completed, the seven items were averaged together to create a summary score, and then transformed to a 100-point scale. Positive HRQL was coded as closer to 100.

**Table 1:** **Global HRQL items for parent proxy report**^**a**^

**Question**	**Response Option**
During the past week, how would you rate the general quality of your child’s life in each of the following areas?	Excellent Very good Good
a. Physical health	Fair
b. Mental health	Poor
c. Family life	
d. Friendship	
e. Self-confidence	
f. Fun	
g. Life enjoyment	

^a ^Wording for the adolescent self-report items replaces the phrase “your child’s” with “your”.

#### Demographic and medical information

As part of the baseline assessment in both studies, demographic variables on the patients and their parents were obtained using the parent demographic form of the CHRIs. Trained research staff collected clinical data from medical records, including the following baseline variables: transplant type (autologous, related allogeneic, unrelated allogeneic), causal diagnosis (malignancy vs. non-malignancy), and duration of illness in months. The natural logarithm of duration of illness was used because it was highly skewed.

Data on clinical outcomes were also obtained from the medical chart at the end of transplant hospitalization and at each follow-up time by trained research staff. Acute graft versus host disease (aGVHD) and transplant toxicity (as measured by the Bearman toxicity scale) [12] were collected at the end of hospitalization, 45 days, and 3 months. Chronic graft versus host disease (cGVHD) was collected at the end of hospitalization, 3, 6, and 12 months. Infection within the previous week was collected at all time points, using the Common Toxicity Criteria of Adverse Events, v. 3.0. A dichotomous composite variable was created to indicate complications during the first 3 months post-HSCT (hereafter “early complications”). Early complications was defined as experiencing at least one of the following: aGVHD of grade 2 or higher, intermediate or poor toxicity (indicating intermediate or high levels of toxicity), or localized or systemic infection. A separate dichotomous variable was created to indicate the presence of limited or extensive cGVHD between month 6 and 12.

### Statistical analysis

Demographic and clinical variables were summarized separately for parents of all children aged 5–18 and adolescents aged 13–18 using means (standard deviations (SD)) or medians (25th-75th percentiles) for continuous variables or using frequencies and percentages for categorical variables.

#### Scale construction and psychometric properties at baseline

Scale construction and psychometric analyses used baseline data separated into three groups: (1) parent proxy report for all children (aged 5–18), (2) parent proxy report for adolescents (aged 13–18), and (3) adolescent self-report. Principal components analysis was used to determine if the global HRQL scale was unidimensional. We examined scree plots to identify the number of principal components, as indicated by eigenvalues of at least 1.00, and then estimated factor solutions. When identifying principal components, each item should have a factor loading ≥ .40. The amount of variation explained by the component(s) selected was also reported. Cronbach’s alpha was calculated to estimate the internal consistency reliability of the global HRQL scale [13]. The minimum acceptable criterion for Cronbach’s alpha in exploratory scale development is .70, whereas for established scales, Cronbach’s alpha should exceed .80 [14]. Means, *SD*s, ceiling and floor effects, and percent missing were calculated for each item and the global HRQL scale score.

#### Repeated measures model to explore global HRQL trajectory over time

In addition to exploring global HRQL trajectories, these models also allow us to begin establishing the construct validity of the scale. Two separate repeated measures models were constructed: (1) global HRQL for all children based on parent proxy report (“all children” model) and (2) global HRQL for adolescents based on adolescent self-report and parent proxy report (“adolescent only” model). The outcome variable was the global HRQL score, as measured by the CHRIs, at baseline, 45 days, and 3, 6, and 12 months. Given the non-linear relationship between time and the outcome, time was treated as a categorical variable with baseline as the reference. We used maximum likelihood estimation with repeated measures in SAS Proc Mixed to account for the correlations over time, with an unstructured covariance matrix. Univariate analysis was done to determine which variables to potentially include in the multivariable models and variables with *p* > 0.1 were then removed using backwards elimination. For the “all children” model, the following variables and interaction terms were considered: baseline parental emotional functioning, child age, parent gender, child gender, baseline timing, log duration of illness, transplant type, early complications, late cGVHD, time interacted with (hereafter noted by *) baseline parental emotional functioning, time*baseline timing, time*early complications, and time*late cGVHD. In addition to the variables in the “all children” model, the “adolescent only” model also considered an indicator for rater (parent or child), time*rater, baseline parent emotional functioning*rater, parent gender*rater, and child age*rater. Baseline parental emotional functioning was included because it has been shown to be associated with the child’s HRQL [15,16]. Linear and quadratic forms of child age were tested. Baseline timing was an indicator for whether or not the baseline HRQL assessment was completed before or during the child’s preparative regimen; our previous research shows that those completing assessments during the preparative regimen reported lower HRQL scores [17]. We chose to build a separate model for adolescents because self-ratings were not available in younger children.

To address the possibility that outcome data may have been missing not at random (MNAR), we stratified the analysis by the extent of missing data, defining strata as follows: (1) those with any missing data due to medical reasons and (2) those with complete data or those with missing data due to non-medical reasons. The stratified models (called pattern mixture models (PMM)) [18] assume the data are missing at random (MAR) within strata. We compared stratified to unstratified models using likelihood ratio tests (LRT).

All analyses were done in SAS version 9.2 (SAS Institute, Inc. Cary, NC, USA); the alpha-level was set at 0.05.

## Results

There were 312 parent proxy reports and 117 adolescent self reports (see Table 2). Parents had a mean age of 40 (*SD*=7) and were predominately mothers (84%). Children and adolescents combined had a mean age of 11 (*SD*=4) and were 51% female. Unrelated allogeneic transplant was the most common transplant type (51%) and 76% of children and adolescents had a malignancy as their causal diagnosis. Over the 12-month HSCT course, 34% of children and adolescents had early complications, while 4% had late cGVHD.

**Table 2:** Baseline demographic and clinical characteristics for parent proxy report and adolescent self-report

	**Mean ( *SD *), *n *(%), or median (25th to 75th percentile)**
Parent proxy report, *n*=312	
Parent age, mean (*SD*)	39.8 (7.1)
Parent female, *n* (%)	261 (83.7%)
Baseline parental emotional functioning, mean (*SD*)	49.9 (19.2)
Child age, mean (*SD*)	10.9 (4.2)
Child female, *n *(%)	158 (50.6%)
Transplant Type	
Autologous	70 (22.4%)
Allogeneic, related	84 (26.9%)
Allogeneic, unrelated	158 (50.6%)
Causal Malignancy, *n *(%)	236 (75.6%)
Duration of illness, median (25th to 75th)	11.0 (5.0, 40.0)
Study, *n *(%)	
JTR	164 (52.9%)
HSCT-CHESS™	147 (47.1%)
Baseline measures during preparative regimen, *n *(%)	96 (30.8%)
Adolescent self-report, *n*=117	
Child age, mean (*SD*)	15.6 (1.8)
Child female, *n *(%)	54 (46.2%)
Transplant Type, *n *(%)	
Autologous	28 (23.9%)
Allogeneic, related	31 (26.5%)
Allogeneic, unrelated	58 (49.6%)
Causal Malignancy, *n *(%)	96 (82.1%)
Duration of illness, median (25th to 75th)	12.0 (5.0, 34.0)
Study, *n *(%)	
JTR	59 (50.4%)
HSCT-CHESS™	58 (49.6%)
Baseline measures during preparative regimen, *n *(%)	39 (33.3%)

Abbreviations: *SD *standard deviation, *JTR *Journeys to Recovery, *HSCT-CHESS*™ Hematopoietic Stem Cell Transplant-Comprehensive Health Enhancement Support Study (CHESS™).

### Scale construction and psychometric properties at baseline

Principal components analysis indicated that the global HRQL scale was unidimensional for all three groups: (1) all children parent proxy report, (2) adolescent only parent proxy report, and (3) adolescent self-report. The scree plots for each of the three different groups indicated only one component with an eigenvalue greater than 1.0 and all factor loadings exceeded .40. The amount of variation explained by the single component was 62% for parent proxy report of all children, 64% for parent proxy report of adolescent, and 45% for adolescent self-report. Cronbach’s alpha was .90 for parent proxy report of all children, .91 for parent proxy report of adolescents, and .80 for adolescent self-report. Item- and scale-level psychometrics are presented in Table 3. Of note, the physical health item had the lowest mean score of all the items. Variability was similar across items and between raters and there was no indication of strong ceiling or floor effects. There was very little missing data for each item (< 2%).

**Table 3:** Baseline item and scale psychometrics

	**All Children (5–18 years)**				**Adolescents Only (13–18 years)**							
	**Parent Proxy Report (n=312)**				**Parent Proxy Report (n=117)**				**Adolescent Self-Report (n=117)**			
	**Mean ( *SD *)**	**% Floor**	**% Ceiling**	**% Missing**	**Mean ( *SD *)**	**% Floor**	**% Ceiling**	**% Missing**	**Mean ( *SD *)**	**% Floor**	**% Ceiling**	**% Missing**
Global Scale	61.2 (21.9)	0.0	4.5	0.3	58.9 (22.0)	0.0	3.4	0.0	69.0 (18.2)	0.0	2.6	0.9
Items												
Physical health	54.0 (26.7)	5.2	11.3	0.6	50.6 (26.3)	5.2	9.5	0.9	58.2 (27.1)	6.0	15.5	0.9
Mental health	60.6 (28.4)	2.9	20.9	0.3	57.5 (28.9)	4.3	18.0	0.0	69.6 (25.7)	1.7	29.3	0.9
Family life	65.8 (27.2)	1.9	25.4	0.3	64.7 (26.3)	1.7	23.1	0.0	75.0 (24.3)	0.0	38.3	1.7
Friendships	60.0 (30.5)	8.4	21.9	0.6	60.5 (30.5)	8.6	23.1	0.0	73.9 (29.1)	4.4	44.4	1.7
Self-confidence	67.4 (25.1)	1.3	23.5	0.3	66.2 (25.5)	1.7	21.4	0.0	72.0 (24.3)	1.7	31.0	0.9
Fun	60.0 (28.8)	7.1	19.0	0.3	57.3 (27.9)	6.0	15.4	0.0	68.5 (29.4)	6.1	33.9	1.7
Life Enjoyment	60.4 (28.2)	3.9	19.6	0.3	55.6 (26.9)	5.1	11.1	0.0	66.4 (27.6)	6.0	25.0	0.9

Abbreviations: *SD *standard deviation.

### Repeated measures model to explore global HRQL trajectory over time

#### All children model results

**Figure 1:**
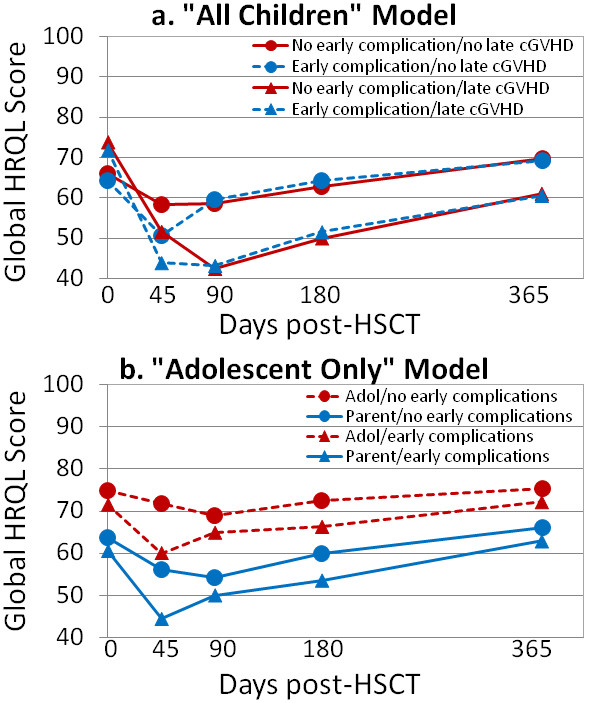
**Global HRQL means scores for “All Children” and “Adolescent Only” based on pattern mixture models. **Abbreviations: HRQL = health-related quality of life; HSCT = hematopoietic stem cell transplant; Adol = adolescent. Notes: Early complications refers to at least one of the following within the first 3 months: aGVHD of grade 2 or higher, intermediate or poor toxicity, or localized or systemic infection. Late cGVHD refers to limited or extensive cGVHD between 6 and 12 months.

The following covariates and interaction terms were significant in univariate analysis and were not removed from the “all children” multivariable model during backwards elimination: time, baseline parental emotional functioning, child age, baseline timing, log duration of illness, transplant type, early complications, late cGVHD, time*baseline timing, time*early complications, and time*late complications. The LRT comparing the repeated measures model to the PMM revealed the presence of MNAR by medical missingness (*χ*^*2*^ [*df* = 25] = 168, *p* < .0001); model estimates, standard errors (SE), and p-values presented in the results section, Table 4, and Figure 1 are from the PMM. For children with baseline measures completed before the preparative regimen and without early complications or late cGVHD, parent proxy report of global HRQL scores were significantly lower at 45 days (*β* = -8.5, *SE* = 2.4, *p* = .0003) and 3 months (*β* = -8.0, *SE* = 2.6, *p* = .002) than baseline. Children with baseline measures completed during the preparative regimen had baseline scores that were 12 points lower than those with measures completed before the regimen (*β* = -11.8, *SE* = 2.7, *p* < .0001), but this difference in scores diminished over time (see Table 4 for coefficients and p-values for the time*baseline timing interaction). Parents with baseline parental emotional functioning scores that were half a standard deviation higher (1/2 *SD* = 9.5) reported their child’s global HRQL as 3.8 points higher (*SE* = 0.4, *p* < .0001) than parents with lower baseline emotional functioning scores. An increase in one year of child age was associated with global HRQL scores that were half a point lower (*SE* = 0.2, *p* = .008) than younger children. Longer log duration of illness was marginally associated with lower global HRQL scores (*β* = -1.2, *SE* = 0.7, *p* = .07). Compared to allogeneic unrelated donor transplants, children with autologous or allogeneic related donor transplants had higher global HRQL scores over time (*β* = 4.2, *SE* = 2.2, *p* = .06 and *β* = 4.7, *SE* = 2.0, *p* = .02, respectively). Children with early complications experienced a marginally significant 6-point drop in global HRQL from baseline to 45 days (*β* = -6.4, *SE* = 3.4, *p* = .06). The presence of late cGVHD was associated with decreased global HRQL at 3 months (*β* = -21.9, *SE* = 9.7, *p* = .02) and 6 months (*β* = -20.0, *SE* = 9.4, *p* = 0.03) compared to baseline. Mean trajectories were plotted to compare global HRQL scores by the presence of early complications or late cGVHD (Figure 1a).

**Table 4:** Multivariable pattern mixture models

	**“All Children” Model**		**“Adolescent only” Model**	
	***β *( *SE *)**	***p*-value**	***β *( *SE *)**	***p*-value**
Intercept	49.7 (4.1)	<.0001	90.3 (13.5)	<.0001
Time				
Baseline (reference)				
45 days	-8.5 (2.4)	.0003	-3.2 (3.4)	0.35
3 months	-8.0 (2.6)	.002	-6.0 (3.7)	0.11
6 months	-4.8 (2.6)	.07	-3.5 (4.1)	0.39
12 months	2.0 (4.8)	.68	-0.1 (5.1)	0.98
Baseline timing	-11.8 (2.7)	<.0001	-14.3 (3.4)	<.0001
Time*Baseline timing				
Baseline*baseline timing (reference)				
45 days*baseline timing	7.5 (3.4)	.03	8.6 (4.7)	0.06
3 months*baseline timing	6.8 (3.7)	.07	9.7 (4.9)	0.048
6 months*baseline timing	11.5 (3.7)	.002	11.7 (5.3)	0.03
12 months*baseline timing	9.6 (5.8)	.10	8.8 (6.3)	0.16
Baseline parental emotional functioning for ½ *SD *change^a^	3.8 (0.4)	<.0001	2.2 (0.7)	0.002
Baseline parental emotional functioning*parent rater for ½ *SD* change^a^			1.7 (0.6)	0.003
Child age	-0.5 (0.2)	.008	-1.7 (0.8)	0.03
Log duration of illness	-1.2 (0.7)	.07		
Transplant Type				
Autologous	4.2 (2.2)	.06		
Allogeneic, related	4.7 (2.0)	.02		
Allogeneic, unrelated (reference)				
Early complications^b^	-1.8 (2.7)	.49	-2.0 (3.4)	0.57
Time*early complications^b^				
Baseline*early complications (reference)				
45 days*early complications	-6.4 (3.4)	.06	-10.1 (4.7)	0.03
3 months*early complications	2.3 (3.6)	.52	-2.2 (4.9)	0.66
6 months*early complications	4.1 (3.8)	.27	-3.3 (5.4)	0.54
12 months*early complications	0.3 (6.2)	.96	-1.2 (6.3)	0.85
Late cGVHD^c^	7.6 (7.7)	.32		
Time*Late cGVHD^c^				
Baseline*late cGVHD (reference)				
45 days* late cGVHD	-12.5 (9.3)	.18		
3 months* late cGVHD	-21.9 (9.7)	.02		
6 months* late cGVHD	-20.0 (9.4)	.03		
12 months* late cGVHD	-14.3 (10.5)	.17		
Parent Female			-1.5 (4.0)	0.71
Parent Rater			-13.3 (4.6)	0.004
Parent female*Parent rater			-6.6 (3.2)	0.04
Time*Parent rater				
Baseline*parent rater (reference)				
45 days*parent rater			-4.5 (2.4)	0.054
3 months*parent rater			-3.3 (2.4)	0.18
6 months*parent rater			-1.5 (2.7)	0.57
12 months*parent rater			1.1 (2.9)	0.71

Abbreviations: *SE *standard error, *SD *standard deviation, *cGVHD* chronic graft versus host disease.

^a ^A half standard deviation for the parent emotional functioning is equal to 9.5.

^b ^Early complications refers to at least one of the following within the first 3 months: aGVHD of grade 2 or higher, intermediate or poor toxicity, or localized or systemic infection.

^c^ Late cGVHD refers to limited or extensive cGVHD between 6 and 12 months.

#### Adolescent only model results

For the “adolescent only” multivariable model, the following covariates and interaction terms were included based on univariate analysis and not removed during backwards elimination: time, baseline parental emotional functioning, child age, parent gender, rater, baseline timing, early complications, time*rater, parent gender*rater, time*baseline timing, rater*baseline parent emotional functioning, and time*early complications. Similar to the “all children” model, the LRT comparing the “adolescent only” repeated measures model to the PMM also revealed the presence of MNAR by medical missingness (*χ*^*2*^ [*df* = 25] = 135.8, *p* < .0001); model estimates, SE, and p-values are from the PMM (see Table 4 and Figure 1). Among adolescents with baseline measures completed before the preparative regimen and with no early complications, both raters did not report a change in global HRQL over time (see estimates for time in Table 4). When baseline measures were completed during the preparative regimen, global HRQL scores were reported as 15 points lower (*SE* = 3.4, *p* < .0001) compared to those completing the measures before the preparative regimen, but this difference in scores diminished somewhat over time (see Table 4 for coefficients and p-values for the time*baseline timing interaction). Adolescent children of parents with baseline parental emotional functioning that was a half standard deviation higher (1/2 *SD* = 9.5) reported their own global HRQL as 1.9 points higher (*SE* = 1.0, *p* = .003) compared to adolescent children of parents with lower emotional functioning scores, while their parents reported their child’s global health an additional 1.9 points higher (*SE* = 1.0, *p* = .001) compared to parents with lower emotional functioning scores. An increase in one year of child age was associated with global HRQL scores that were 1.5 points lower (*SE* = 0.7, *p* = .04) compared to younger children. At baseline, parents rated their adolescent child’s global HRQL as 13.3 points lower than the adolescent’s self-report (*SE* = 4.6, *p* = .004) and this difference increased by nearly 5 points at 45 days (*β* = -4.5, *SE* = 2.4, *p* = .054). For adolescents, there was no difference in global HRQL scores by parent gender (*β* = -1.5, *SE* = 4.0, *p* = .71), but mothers rated their adolescent child’s global HRQL 6.6 points lower than fathers (*SE* = 3.2, *p* = .04). Among adolescents who experienced early complications, both raters reported statistically significant decreases in global HRQL at 45 days compared to baseline scores (*β* = -10.1, *SE* = 4.7, *p* = .03). Mean trajectories were plotted to compare global HRQL scores by rater and the presence of early complications (Figure 1b).

## Discussion

This study has demonstrated the unidimensionality of a 7-item global HRQL scale in a sample of children undergoing HSCT. Both parent proxy report and adolescent self-report showed acceptable levels of internal consistency reliability, little missing data, and similar item variability. Additionally, we explored the trajectories of global HRQL in children over the 12-month clinical course following HSCT, which helps to establish this scale’s construct validity.

This global HRQL scale, the first for use in a pediatric population, contains items about the following areas: physical health, mental health, family life, friendships, self-confidence, fun, and life enjoyment. All three reports (all children parent proxy report, adolescent only parent proxy report, and adolescent self-report) of the global HRQL items indicated that the child’s illness had the most impact on their physical health as reflected by the lowest mean item score. The Cronbach’s alpha coefficients for parent proxy report were at least 0.90, while adolescent self-report was slightly lower (0.80). Although the Cronbach’s alpha for the adolescent self-report is still above the minimum acceptability criterion for exploratory scale development [14], other HRQL scale development research has also shown lower levels of internal consistency reliability in child self-report than parent proxy report [4,19]. Principal components analysis indicated that more than 60% of the variation was explained by the single component for parent proxy report, but only 45% of variation was explained for adolescent self-report. Similar to lower Cronbach’s alphas in adolescent self-report, this decreased variation explained could also reflect differences in scale performance by parent proxy and adolescent self-report and warrant further study.

The results of the principal components analysis, including the scree plot and factor loadings, confirmed the presence of a unidimensional global HRQL scale. In contrast to multi-dimensional pediatric HRQL measures, this global HRQL scale can decrease respondent burden, increase ease of implementation, increase simplicity by having a single outcome, and direct future research and evaluation. Although the unidimensional nature of the scale may reduce the ability to discern which areas of functioning are affected, looking at individual item scores could provide additional information. This measure could be completed outside of clinic to assess changes over time or could be used prior to a clinic visit to begin dialogue about the child’s health and HRQL. In addition, it could serve as a screener for either further HRQL assessment or clinical intervention. In parallel work in adults, using the PROMIS global scale, mapping has been done with the EuroQol 5 Dimension (EQ-5D) [11], which is a preference-based instrument that generates a utility weight, which can be used in cost-effectiveness or comparative effectiveness studies.

In both the “all children” and “adolescent only” models, higher parent emotional functioning scores were associated with higher global HRQL scores in children. When rating the child’s HRQL, parents with lower parental emotional functioning may perceive their child’s global HRQL as lower. Although lower parental emotional functioning could cause children and adolescents to experience lower global HRQL themselves [15,16], the significant interaction between rater and baseline emotional functioning in the “adolescent only” model indicates a larger effect on the parent rater than the adolescent rater. Older child age was associated with lower global HRQL scores potentially reflecting cognitive and maturity differences in how older children perceive their illness. In both models, the presence of early complications, such as aGVHD, toxicity, and infection, was associated with decreased global HRQL within the early phases of the post-HSCT course (45 days and 3 months). Additionally, the “all children” model showed decreased global HRQL for children with late cGVHD within the later phases of the post-HSCT course (3 and 6 months). Given that only 7 adolescents among the 117 included in the adolescent model had late cGVHD, we were likely underpowered to detect any differences in the “adolescent only” model. These decreases in the global HRQL trajectories based on clinical outcomes establishes construct validity of the scale. For those who completed the measures during the preparative regimen, global HRQL scores were lower at baseline. These differences diminished over time in the “all children” and “adolescent only” models.

For the “adolescent only” model, parent proxies reported lower baseline global HRQL scores than adolescent self-report. Inter-rater differences between parent proxies and adolescents have several possible explanations, including information variance and criterion variance. Information variance could occur when the parent and child receive different information about clinical events, while criterion variance could occur when they weigh and respond to available information differently. These rater differences illustrate the importance of collecting pediatric HRQL data from both the parent and the child [20]. In addition, we found that fathers rated their adolescent child’s global HRQL higher than mothers. Previous research has shown that the child’s illness causes different levels of parental distress in mothers and fathers [21], which in turn may affect how they rate their child’s HRQL [22].

This study has several limitations. At this time, self-reported global HRQL items were only completed by adolescents, not from children aged five to twelve, thus preventing us from comparing ratings in younger children. Future testing is needed to determine how children aged 8–12 are able to respond to the global items and separately estimate the psychometric properties within this age group. A second limitation is that some participants were lost to follow-up, as is common in longitudinal studies of patients with severe illnesses who are undergoing intense treatment. However, by stratifying the analysis by whether the data were missing for medical reasons using a pattern mixture model, we mitigated the effects of data MNAR.

## Conclusions

Despite these limitations, this study presents a unidimensional pediatric global HRQL scale for parent proxy report and adolescent self-report. Our sample focused on children undergoing HSCT, but future validation is needed in other pediatric populations. The use of a pediatric global HRQL scale will further facilitate the incorporation of patient-reported and patient-centered outcomes into clinical and epidemiological research and will hopefully lead to improved outcomes in pediatric populations.

## Abbreviations

aGVHD: Acute graft versus host disease; cGVHD: Chronic graft versus host disease; CHRIs: Child Health and Ratings Inventories; HRQL: Health-related quality of life; HSCT: Hematopoietic stem cell transplant; HSCT-CHESS: HSCT-Comprehensive Health Enhancement Support Study (CHESS™); JTR: Journeys to Recovery study; MAR: Missing at random; MNAR: Missing not at random; PCORI: Patient-Centered Outcomes Research Institute; PMM: Pattern mixture model; PROMIS: Patient-Reported Outcome Measurement Information System.

## Competing interests

The authors have no competing interests to report.

## Authors’ contributions

The studies that form the basis of the sample for this paper were developed by SKP. Statistical analyses were devised by AMR, NT, and SKP. The first draft of the manuscript was written by AMR and discussed with NT and SKP. All authors contributed to the content. All authors read and approved the final manuscript.

## Participating institutions and site investigators in JTR and HSCT-CHESS Studies

Central Project Staff

**Tufts Medical Center, Boston, MA: **Susan K. Parsons, MD, MRP, Principal Investigator

Site Principal Investigators

**Baylor College of Medicine/Texas Children’s Hospital, Houston, TX: **Lynette Harris, PhD and Robert A. Krance, MD, Principal Investigators

**Children’s Hospital of Philadelphia, Philadelphia, PA: **Lisa Schwartz, PhD, Principal Investigator

**Cincinnati Children’s Hospital Medical Center, Cincinnati, OH: **Stella Davies, MMBS, PhD, Principal Investigator

**City of Hope, Duarte, CA:** Sunita Patel, PhD, Principal Investigator; Joseph Rosenthal, MD, Site Consultant

**Dana-Farber Cancer Institute, Boston, MA: **Christopher Recklitis, PhD, MPH, JTR Principal Investigator; Eva Guinan, MD, HSCT-CHESS Principal Investigator

**Fred Hutchinson Cancer Research Center, Seattle, WA: **Karen L. Syrjala, PhD, Principal Investigator; Jean Sanders, MD, Co-Principal Investigator

**Medical College of Wisconsin, Milwaukee, WI: **Mary Jo Kupst, PhD, Principal Investigator; Kristin Bingen, PhD and James Casper, MD, Co-Principal Investigators

**University of Pittsburgh/Children’s Hospital of Pittsburgh, Pittsburgh, PA: **Robert B. Noll, PhD, Principal Investigator; Linda J. Ewing, PhD, RN, Co-Principal Investigator
